# Supplementary material for: Anti-Inflammatory Constituents of Antrodia camphorata on RAW 264.7 Cells Induced by Polyinosinic-Polycytidylic Acid
Source: Molecules. 2022 Aug 20;27(16):5320. doi: 10.3390/molecules27165320 (PMC9414214; doi:10.3390/molecules27165320)
Supplement: Supplementary file 1 [file molecules-27-05320-s001.zip › molecules-1828108-supplementary.pdf]

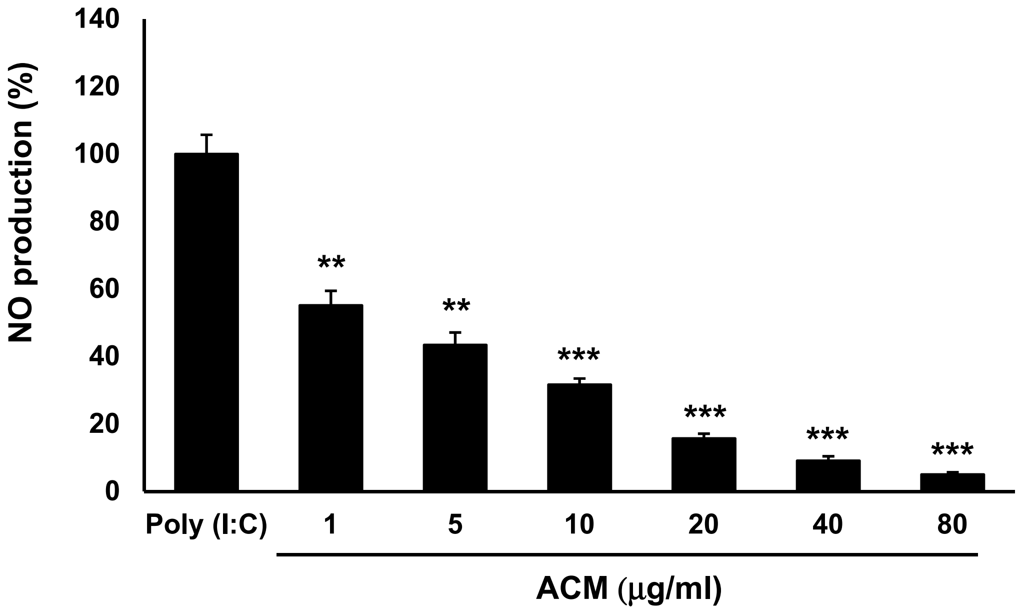

Figure S1. The effects of the mycelium of *Antrodia camphorata* (ACM) on nitric oxide (NO) production in RAW 264.7 cells induced by polyinosinic–polycytidylic acid (poly I:C). Values are the mean ± SD of triplicates. ** $p < 0.01$ *vs.* Con. *** $p < 0.001$ *vs.* Con.

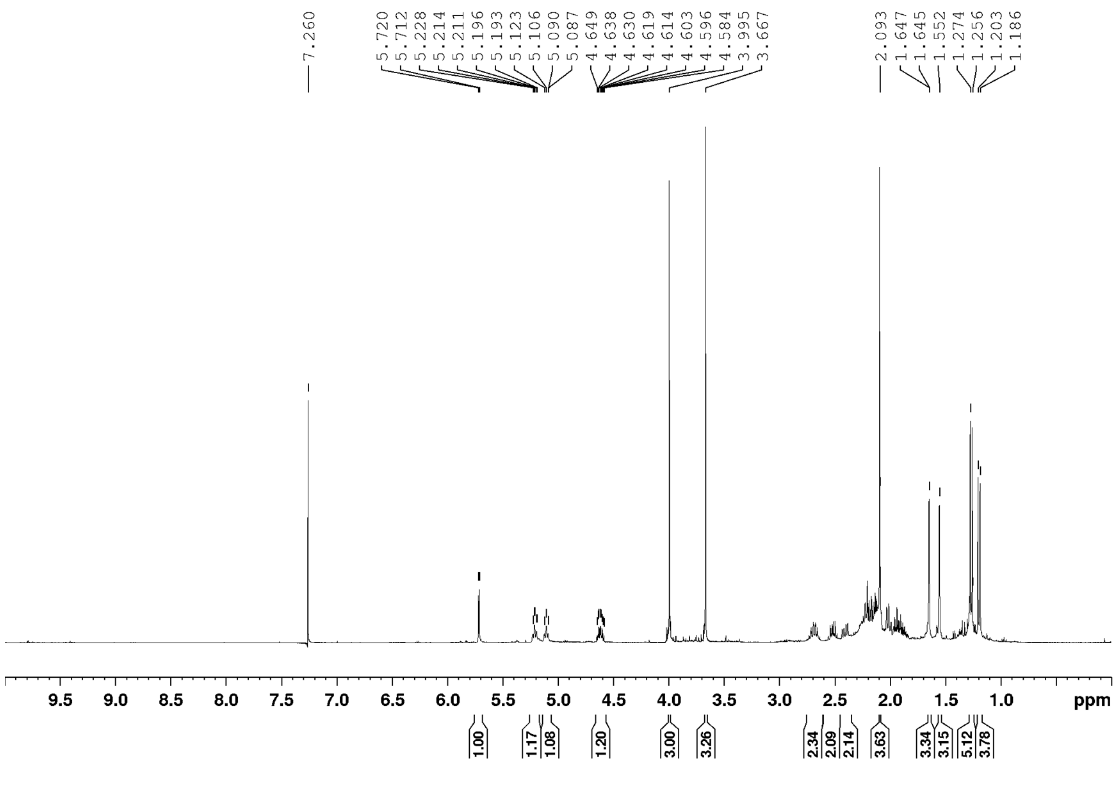

Figure S2. ^{1}H NMR spectrum of the major compound identified as 4-acetylantroquinonol B in the bioactive Fraction 5.

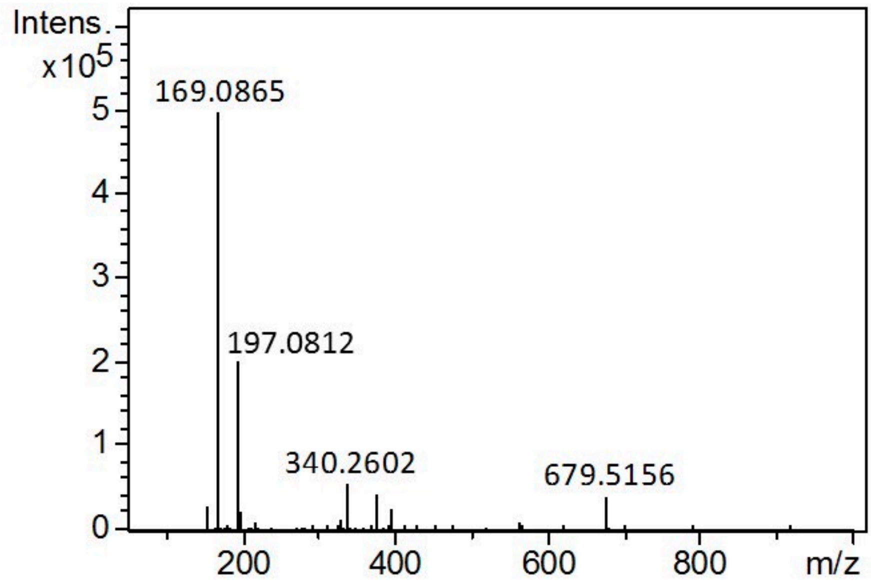

Figure S3. MS spectrum of compound **4**.

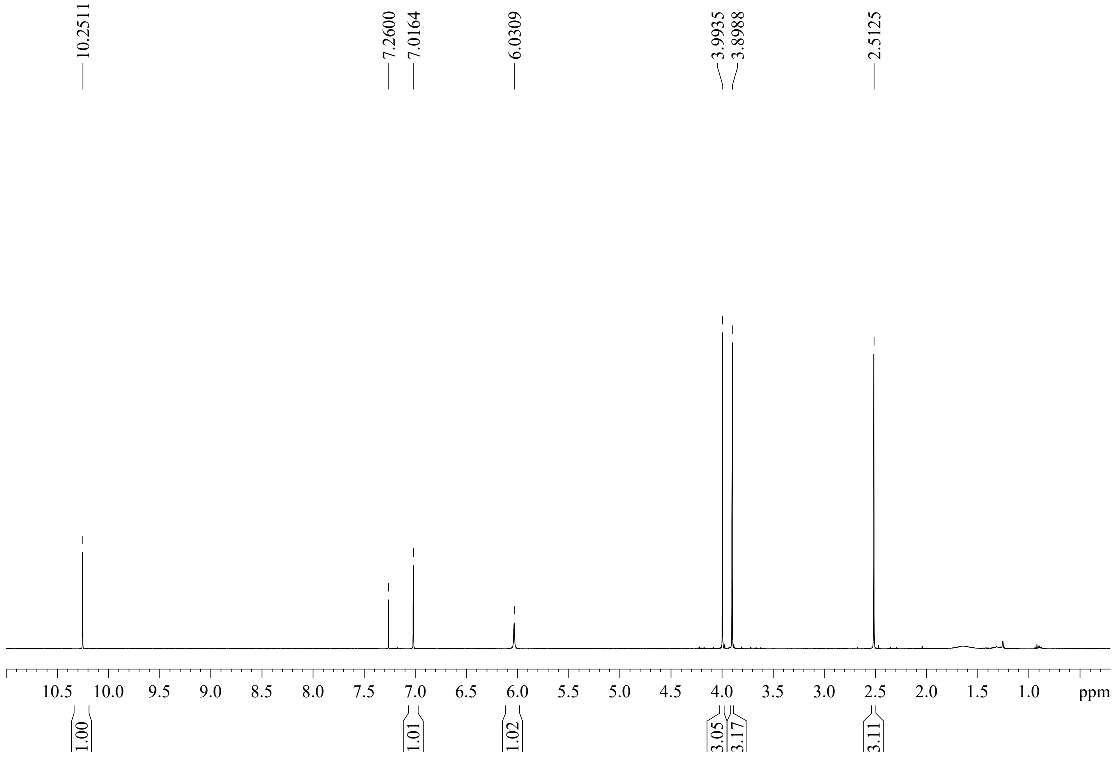

Figure S4. ^{1}H NMR spectrum of compound **4**.

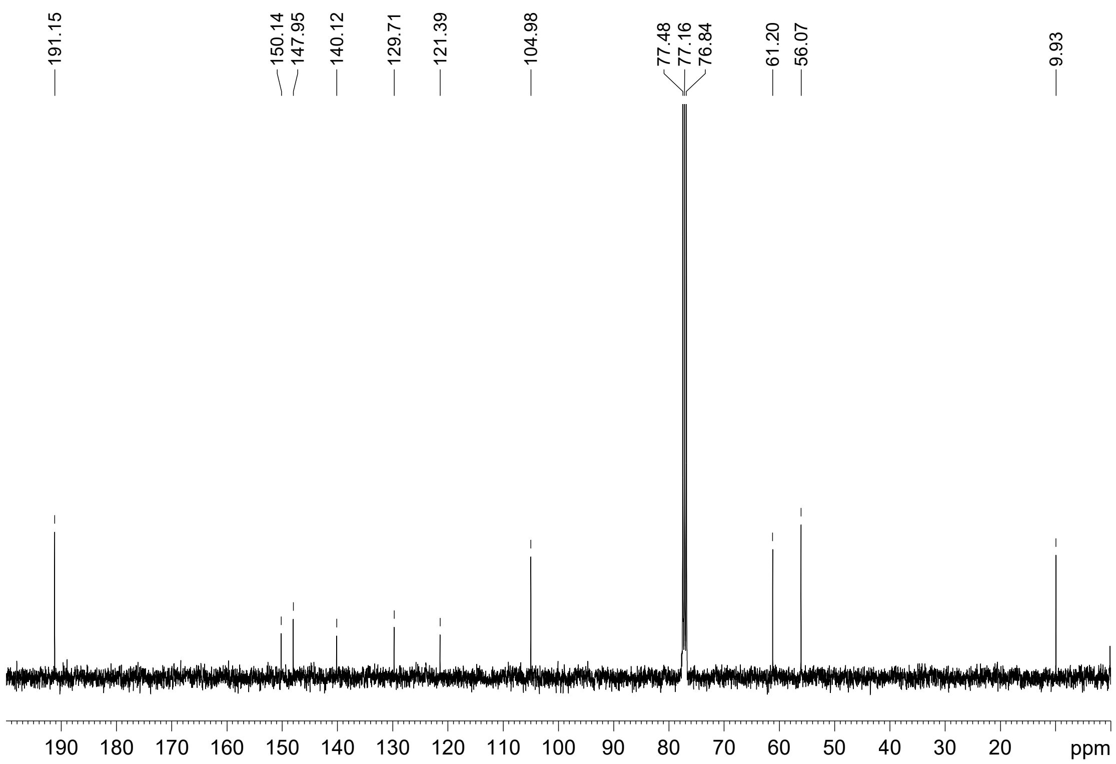

Figure S5. ^{13}C NMR spectrum of compound **4**.

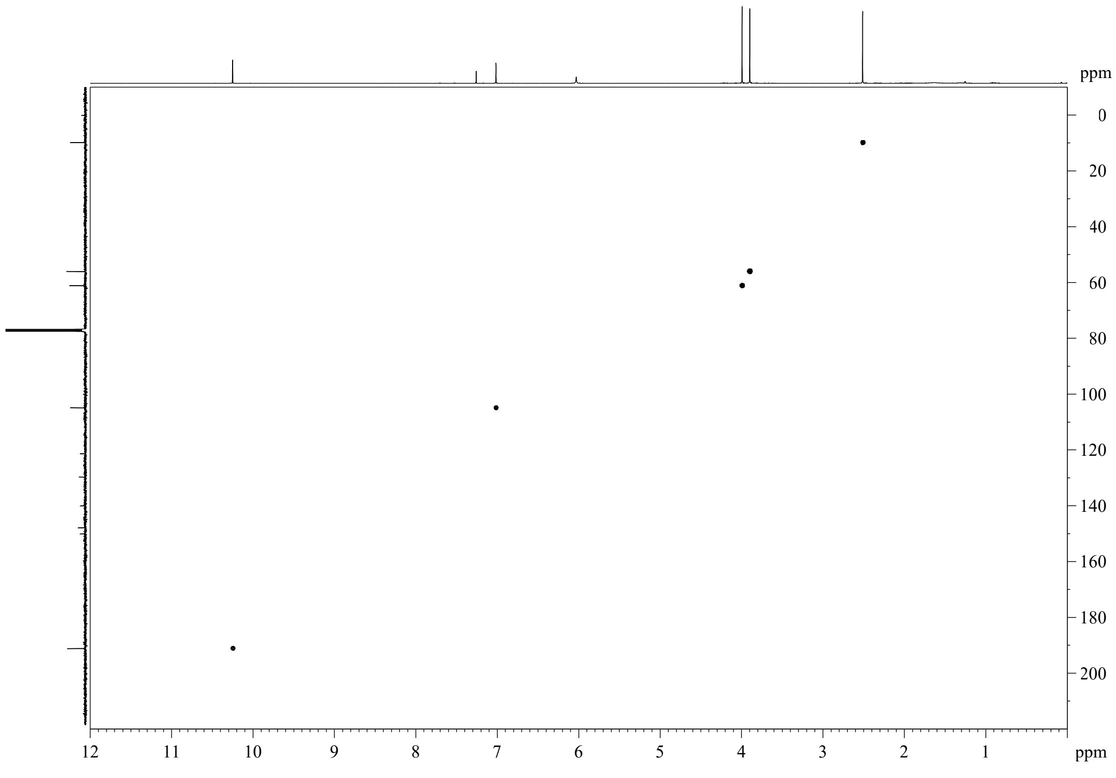

Figure S6. HSQC NMR spectrum of compound **4**.

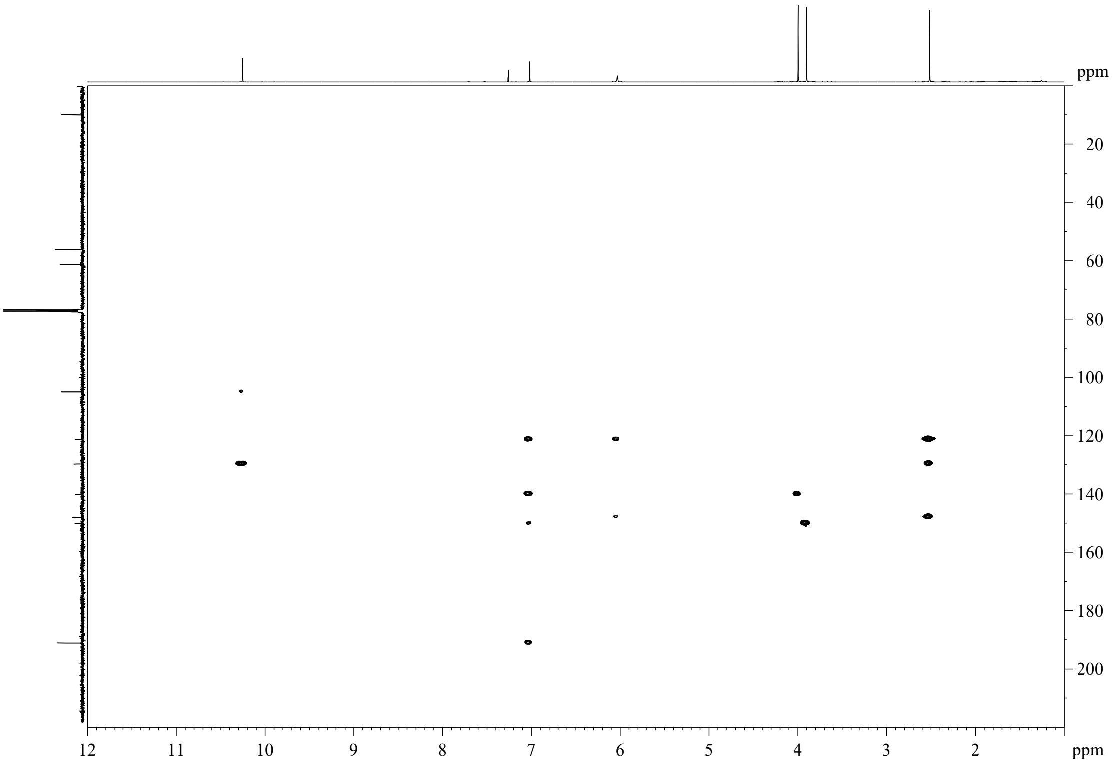

Figure S7 HMBC NMR spectrum of compound **4**.

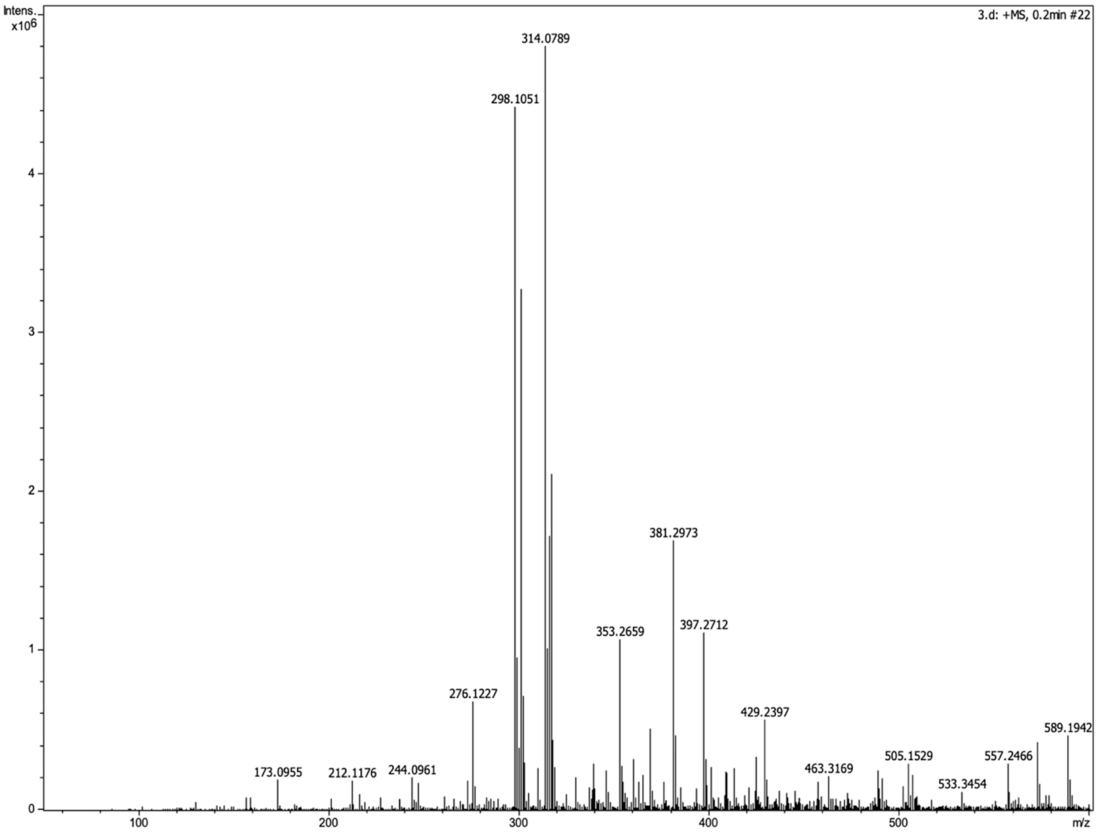

Figure S8 MS spectrum of compound **5**.

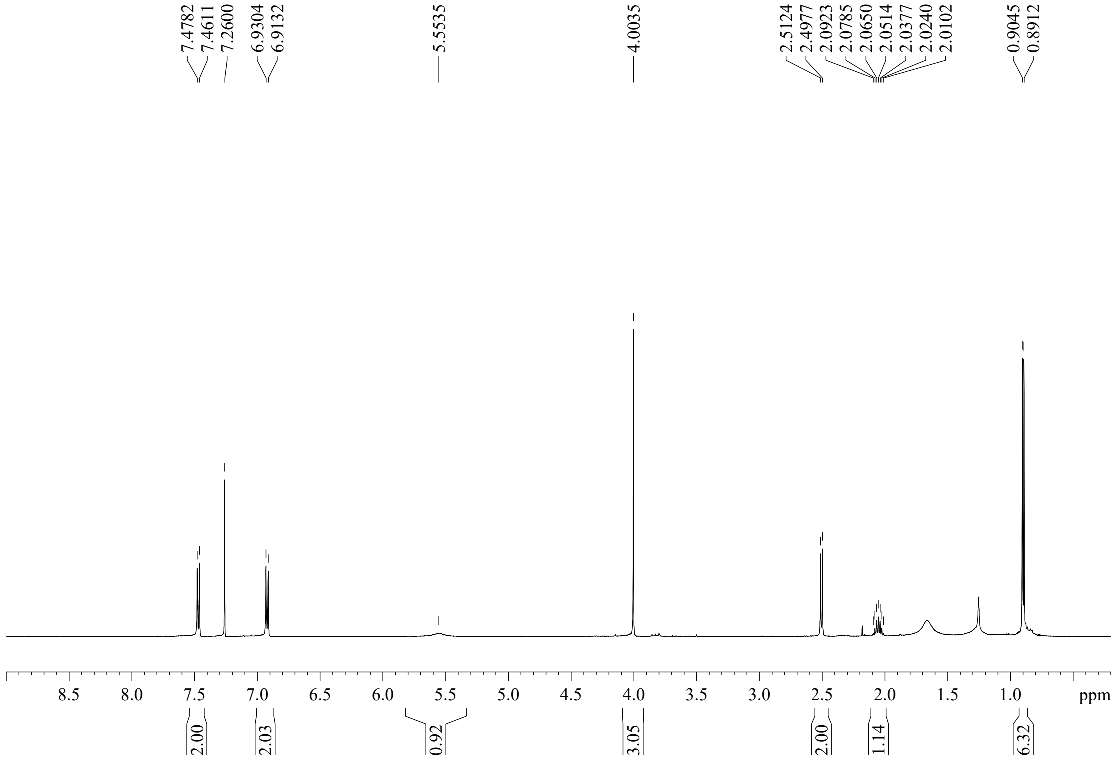

Figure S9 ^{1}H NMR spectrum of compound **5**.

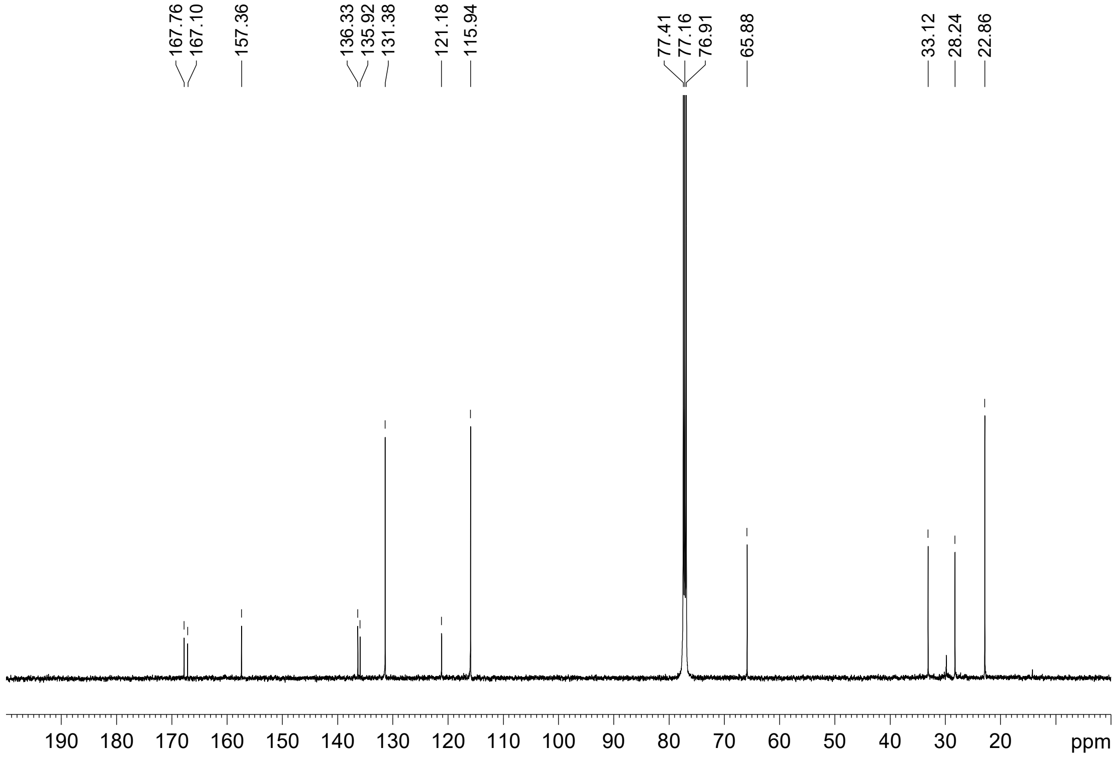

Figure S10 ^{13}C NMR spectrum of compound **5**.

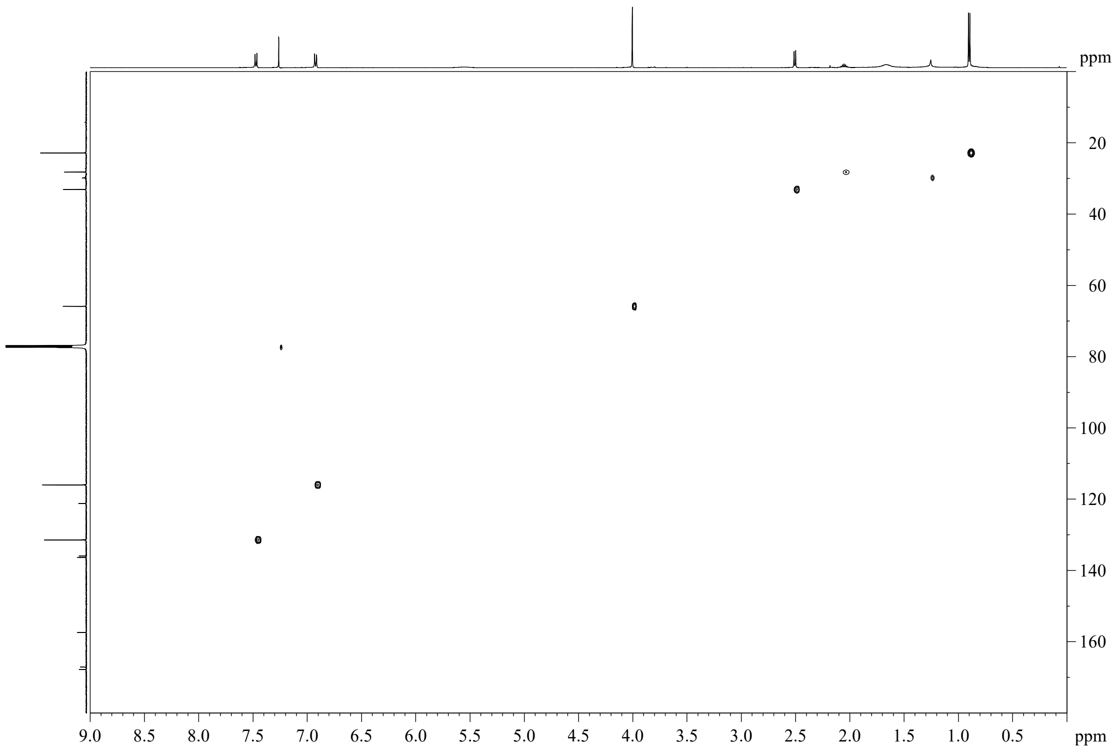

Figure S11 HSQC NMR spectrum of compound **5**.

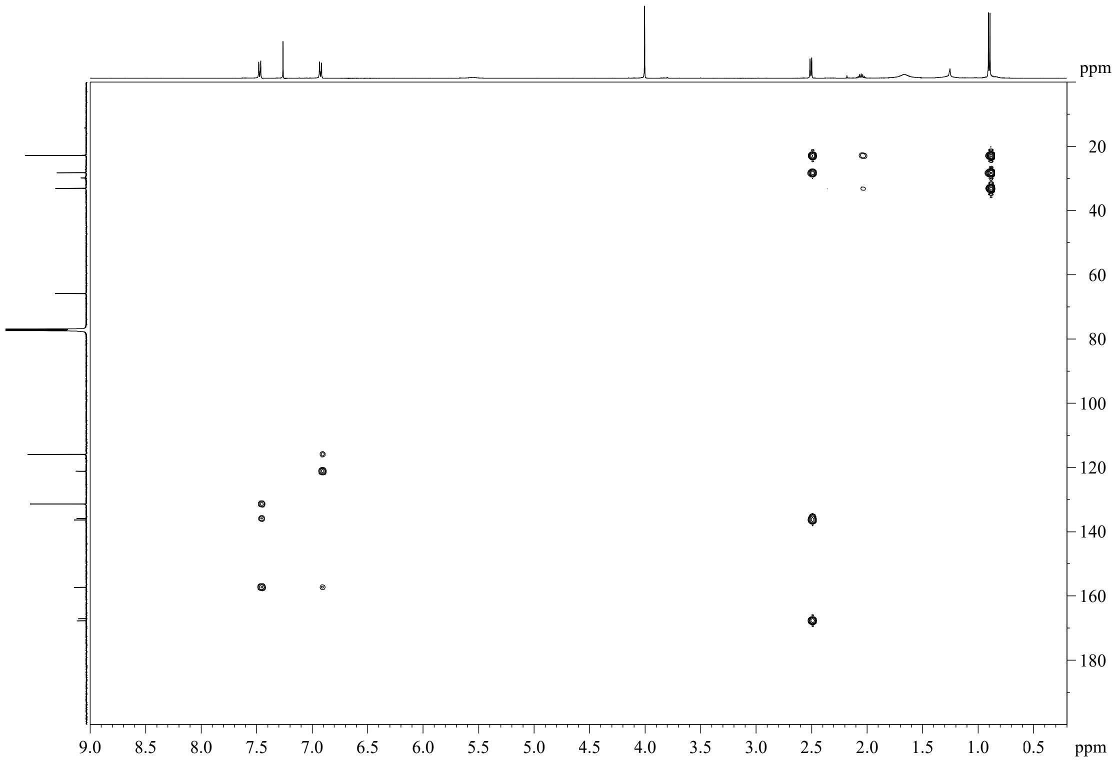

Figure S12 HMBC NMR spectrum of compound **5**.

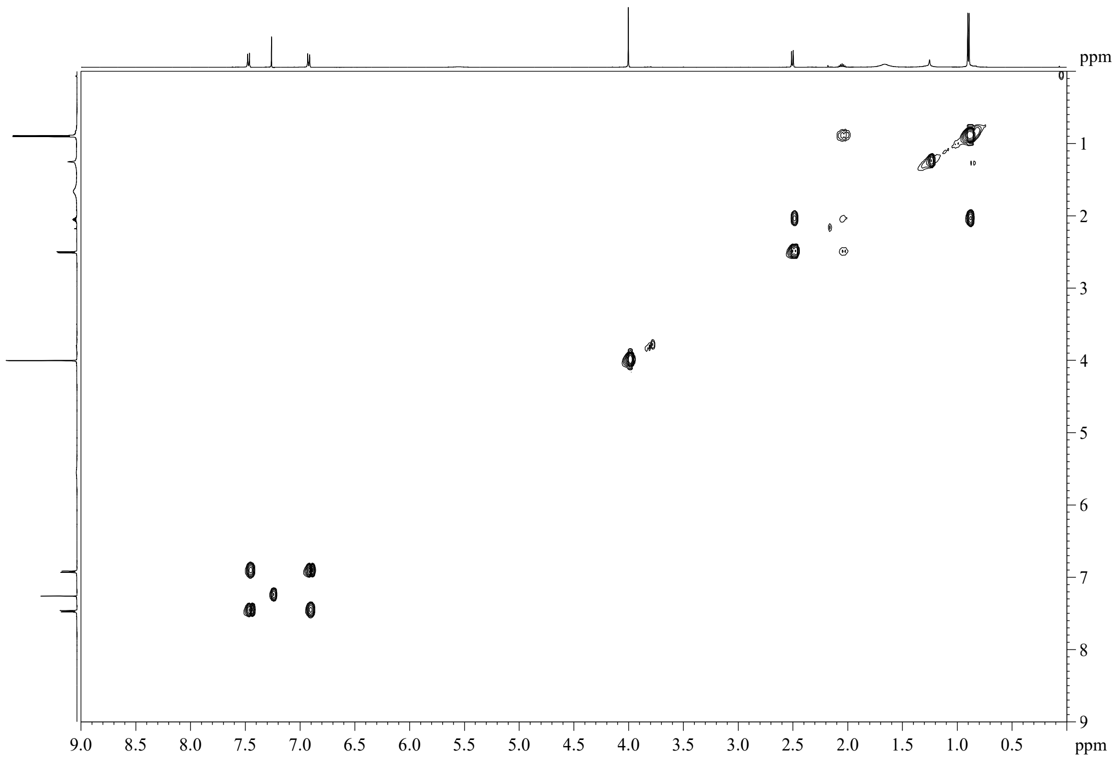

Figure S13 COSY NMR spectrum of compound **5**.
